# Symmetry reduction induced by argon tagging gives access to low-lying excited states of FeH^+^ in the overtone region of the Fe–H stretching mode†

**DOI:** 10.1039/d4cp03270e

**Published:** 2024-10-01

**Authors:** Shan Jin, Marcos Juanes, Christian van der Linde, Milan Ončák, Martin K. Beyer

**Affiliations:** a Institut für Ionenphysik und Angewandte Physik, Universität Innsbruck Technikerstraße 25 6020 Innsbruck Austria milan.oncak@uibk.ac.at martin.beyer@uibk.ac.at; b Departamento Química Física y Química Inorgánica, University of Valladolid Paseo de Belén 7 47011 Valladolid Spain

## Abstract

Iron is the most abundant transition metal in the interstellar medium (ISM), and is thought to be involved in a variety of astrochemical processes. Here, we present the infrared multiple photon dissociation (IRMPD) spectra of Ar_1,2_FeH^+^ and their deuterated isotopologues in the region of 2240–14 000 cm^−1^. The Fe–H overtone stretching mode in ArFeH^+^ and Ar_2_FeH^+^ is observed at 3636 ± 28 cm^−1^ and 3659 ± 13 cm^−1^, respectively. Deuteration shifts these bands to 2618 ± 31 cm^−1^ and 2650 ± 14 cm^−1^ in ArFeD^+^ and Ar_2_FeD^+^, respectively. Additionally, the spectra of Ar_2_FeH^+^ and Ar_2_FeD^+^ feature broad transitions at ∼2200–4000 cm^−1^ and ∼4500–6500 cm^−1^. We assign these bands to electronic transitions from the thermally populated X^5^A_2_/X′^5^A_1_ ground state manifold into the A′^5^B_2_ and B^5^A_1_ states, which we model with multi-reference quantum chemical calculations including spin–orbit coupling. The calculations show that these transitions are symmetry forbidden in FeH^+^ and in the equilibrium geometry of ArFeH^+^/ArFeD^+^, while the zero-point oscillation of the bending mode of the triatomic molecule leads to some oscillator strength. Upon addition of the second argon atom, the transitions become weakly allowed in the equilibrium geometry of Ar_2_FeH^+^/Ar_2_FeD^+^ due to symmetry reduction from *C*_∞v_ to *C*_2v_.

## Abstract 2

The overtone of the Fe–H stretch in ArFeH^+^ is observed by argon tagging, but the low-lying electronic transition lights up only in the non-linear Ar_2_FeH^+^ species.

## Graphical abstract

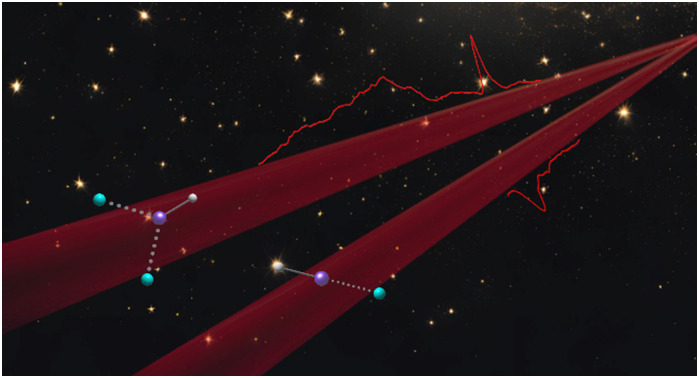

## Introduction

Iron is the most abundant transition metal on Earth, playing an important role in proteins and biochemistry in general.^1^ It has also attracted attention in astrochemical research, in particular due to its abundance in the interstellar medium (ISM).^2–7^ Despite the high abundance of iron, the ISM detection of molecular species containing iron has so far been limited to FeO and FeCN.^2,3^ In the solar system, iron is present in planetary atmospheres as a meteoric ion, with FeH^+^ formation included in the models, *e.g.*, of the ionosphere of Jupiter.^8^ As outlined by E. Dwek^9^ and G. Bilalbegović *et al.*,^10^ iron is potentially a crucial element for understanding interstellar processes and the evolution of interstellar dust. The high abundance of iron in our galaxy together with its limited detection as neutral or ionized gas-phase atom in the ISM is commonly explained by the incorporation of iron in interstellar dust.^11–13^ In support of these arguments, Westphal *et al.* as well as Corrales *et al.* recently reported that ISM X-ray absorption data closely match laboratory spectra of iron oxide/hydroxide minerals.^14,15^ However, although ISM observations show atomic iron to be severely depleted, the recent detection of FeCN in the ISM^3^ or the observed evidence for the presence of FeO in interstellar molecular clouds^2^ shows that iron containing gas-phase molecular species are present in the ISM. Small molecules or complexes containing iron may thus contribute to the hidden iron budget. The previously proven presence of iron in the ISM together with the key role of transition metals in astrophysical environments^16–19^ call for more laboratory work on molecular transition metal compounds.

Since atomic iron is largely ionized in the ISM, and hydrogen is the by far most abundant element, the diatomic FeH^+^ molecular ion has been discussed as a potential iron reservoir species.^20^ A series of quantum chemical studies focused on the electronic structure of FeH^+^ and predicted low-lying electronically excited states.^20–22^ As a first experimental characterization of FeH^+^, we recently studied the vibrational spectrum of Ar_2_FeH^+^ in the 1600–2200 cm^−1^ region.^23^ The Fe–H stretching mode was observed at 1860 cm^−1^, significantly blue-shifted by the argon tag from the 1810.4 cm^−1^ calculated for bare FeH^+^ by Cheng and DeYonker.^20^ Relatively intense combination bands were observed due to the strong anharmonicity of the vibrational modes, which involve argon atoms interacting with the iron center.

Moving to higher photon energies, the next spectral signature of FeH^+^ is expected for the overtone of the Fe–H stretching mode. Due to the small absorption cross sections and difficult quantum chemical modeling, overtone spectroscopy is less frequently used for the identification and characterization of small molecules.^24^ The Metz group observed overtones of ligand bending modes in ammonia complexes of Cr^+^,^25^ while Okumura, Bieske and co-workers observed such transitions in non-covalent complexes of bromide and iodide with ammonia.^26^ Asmis and co-workers recently identified the overtone of the H–H stretching mode in Cu^+^(H_2_)_4_.^27^ Duncan and co-workers reported overtone and combination bands of H_5_^+^ and D_5_^+^.^28,29^ The Dopfer group managed to obtain rotationally resolved overtone spectra of CH_3_^+^–Ar.^30^ Here, we focus on the spectroscopy of ArFeH^+^ and Ar_2_FeH^+^ as well as their deuterated analogues in the 2240–14 000 cm^−1^ region. While we only observe the Fe–H overtone in ArFeH^+^, additional electronic transitions appear in this region for Ar_2_FeH^+^ between the states correlated to the ^5^D states of the iron atom. The absorption cross sections for both vibrational overtone and electronic transitions are on the order of 10^−20^ cm^2^, *i.e.* relatively weak absorptions.

## Experimental and computational methods

The experiments have been performed on a modified Bruker/Spectrospin CMS47X Fourier-Transform Ion Cyclotron Resonance (FT-ICR) mass spectrometer^31,32^ equipped with an external laser vaporization ion source.^33–35^ The title complexes were generated by laser vaporization (frequency-doubled Quantum Light Q2-D33-1053 Nd:YLF laser) of an isotopically enriched iron target, ^56^Fe (99.93%, U.S. DOE), entrained in a supersonic jet expansion of helium carrier gas seeded with argon and hydrogen or deuterium, and guided to the center of the ICR cell, where they are stored and mass selected in a 4.7 T magnetic field.^36^ Typical concentrations are 4% H_2_/D_2_ and 12% Ar in He. In the supersonic expansion into high vacuum, the ionic complexes are cooled to low rotational temperatures. Additionally, the ICR cell is externally cooled with liquid nitrogen, reaching temperatures of *ca.* 80 K, to minimize the contribution of black-body infrared radiative dissociation (BIRD).^37–44^ In our previous work on Ar_2_FeH^+^, we observed some narrowing of the Fe–H stretch in Ar_2_FeH^+^ after waiting for 5 s, which indicates that radiative cooling takes place on a timescale of several seconds and that the ions coming from the source have a vibrational temperature *T*_vib_ > 80 K, most likely closer to room temperature.^23^

Photodissociation spectroscopy is performed by focusing light emitted by a tunable OPO laser system into the ICR cell through a CaF_2_ window.^45^ The infrared light is focused by two lenses with 1.0 m focal length. Tunable monochromatic infrared radiation is generated by an EKSPLA NT277 optical parametric oscillator laser system operating at a 1000 Hz pulse repetition rate, covering the 2240–4000 cm^−1^ region, with typical average laser power of 60–200 mW. The wavelength was calibrated by a HighFinesse Laser Spectrum Analyzer IR-III, which determined the bandwidth as < 1 cm^−1^.^46^ For the 4000–14 000 cm^−1^ range, we used a tunable ESKPLA NT342B optical parametric oscillator laser system with 20 Hz pulse repetition rate, employing the direct output for higher pulse energies, which bypasses the Pellin-Broca prism that is used for wavelength separation in the UV. The wavelength was calibrated by a Flame-S miniature spectrometer (Ocean Optics).

For all complexes, loss of one argon atom was the only photofragmentation channel. To account for laser power and irradiation time, one-photon photodissociation cross sections *σ* are calculated using the modified Beer–Lambert eqn (1).^47^1
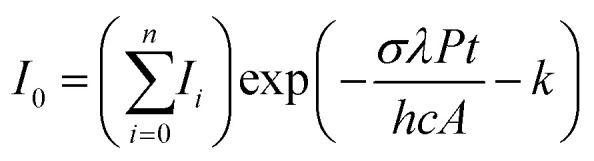
Here, *I*_0_ represents the intensity of the precursor ion, *I*_*i*_, *i* ≥ 1, the fragment ion intensity, *λ* the laser wavelength, *P* the laser power, *t* the irradiation time, *h* Planck's constant, *A* the area of the laser beam and *k* an empirical factor which corrects for the small amount of fragmentation observed without laser irradiation. While one photon is sufficient for loss of argon from Ar_2_FeH^+^, the calculated Ar binding energy indicates that two photons are needed to generate a photodissociation signal in ArFeH^+^. The respective two-photon cross sections are derived with the help of a lookup table as described in detail before.^47^ The major uncertainty of the cross section calculation is the photon flux, which is difficult to determine inside the ICR cell, which is located in the center of the superconducting magnet. We estimate the uncertainty to be within a factor of 2 of the actual values. Band positions and full widths at half maximum (FWHM) are determined by fitting Gaussian line profiles to the spectra using Origin. Throughout the manuscript, we report line positions with an uncertainty of 50% FWHM.

Quantum chemical calculations of structure and vibrational frequencies in the electronic ground state were carried out previously in our work on the spectroscopy of the Ar_2_FeH^+^ fundamental Fe–H stretch.^23^ For the present work, we repeated all calculations with fixed symmetry, slightly changing the calculated vibrational frequencies by 0–3 cm^−1^ compared to previously published values. We also add calculations on the deuterated species. Based on previous benchmarking,^23^ density functional theory (DFT) with various functionals and coupled cluster (CC) approach was used in combination with the aug-cc-pVTZ basis set. As shown elsewhere, the bare FeH^+^ molecular ion has quintet spin multiplicity in the electronic ground state,^20^ and the same holds true for Ar_1,2_FeH^+^.^23^ Very tight convergence criteria were used for geometry optimization. We employed wave function stabilization prior to each calculation. The overtone frequencies calculated using second order vibrational perturbation theory as implemented in Gaussian were again benchmarked using several methods, see ESI,† reusing our previous calculations^23^ where appropriate. Based on the benchmarking and consistent with our previous work,^23^ we report the results on the B3LYP-D3/aug-cc-pVTZ level in the main text. All single-reference calculations were conducted with the Gaussian 16 software package.^48^

Excited electronic states were modeled using the multi-reference configuration interaction (MRCI) approach on the complete active space self-consistent field (CASSCF) calculations. We picked the active space of 8 electrons in 7 orbitals including valence electrons of Fe^+^ (3d^6^4s) as well as the hydrogen electron, further denoted as (8,7). Five electronic states correlating with the ^5^D states of Fe were included in the calculation. Spin–orbit coupling was computed using the Breit–Pauli operator, leading to 25 states in total. For H and Fe, the aug-cc-pVQZ basis set was employed, the ECP10MWB basis set was used for Ar.^49^ This theory level is denoted MRCI(8,7)+SO/aug-cc-pVQZ. The electronic spectra were modeled using the reflection principle,^50–52^ sampling the ground state through 1000 points within Wigner quasiprobabilistic distribution obtained for the vibrational wave function of the complex within harmonic approximation (B3LYP-D3/aug-cc-pVTZ). In ArFeH^+^, we performed two simulations, first with the two stretching vibrations only and then including the degenerate bending vibrations of about 50 cm^−1^ as well. In Ar_2_FeH^+^, we ignored the strongly anharmonic Ar–Fe–Ar bending vibration and, to keep the system computationally tractable, the out-of-plane vibration was removed since it breaks *C*_s_ symmetry. At each point of the sampling, the absorption was broadened using a Gaussian function with the FWHM of 0.03 eV. The modeled spectrum is obtained as the sum of these 1000 Gaussians per electronic transition. We note that this approach provides only semi-quantitative spectra. More advanced methods, *e.g.* path-integral Monte Carlo, would be needed for appropriate ground state sampling. Multi-reference calculations with spin–orbit coupling were performed with Molpro.^53,54^

## Results and discussion

**Fig. 1:**
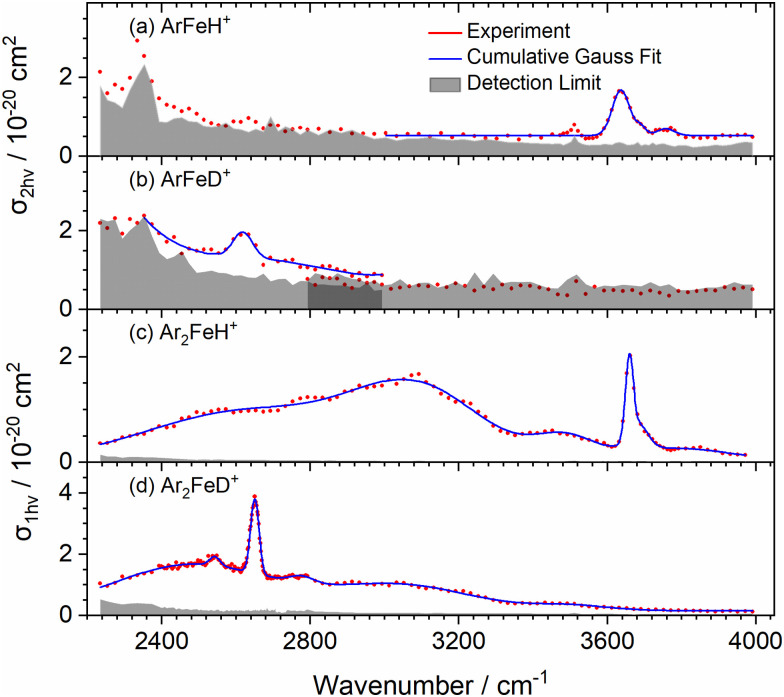
Experimental IRMPD spectra of (a) ArFeH^+^, (b) ArFeD^+^, (c) Ar_2_FeH^+^, and (d) Ar_2_FeD^+^, at *T* ≈ 80 K. (a) and (b) Are evaluated assuming sequential absorption of two photons, (c) and (d) as one photon absorption. The experimental data is presented in red, with cumulative Gauss fits are shown in blue.

We first measured the infrared photodissociation spectra of mass-selected ArFeH^+^, Ar_2_FeH^+^, and their deuterated analogues in the 2240–4000 cm^−1^ region *via* the loss of one argon atom, shown in Fig. 1. The spectrum of ArFeH^+^, Fig. 1a, shows an intense peak at 3636 ± 28 cm^−1^, which is assigned to the overtone of the Fe–H stretching mode. It is accompanied by weaker bands and an unspecific background just above the detection limit. Band positions and widths of the Fe–H/D vibrational overtone transitions are listed in Table 1, determined by fitting Gaussian line profiles to the spectra. Further weak resonances that are sufficiently narrow to be rovibrational transitions are listed in Table S1 (ESI†). ArFeD^+^ has a resonance barely above the detection limit, centered at 2618 ± 31 cm^−1^, Fig. 1b, in the region where the Fe–D stretch overtone is expected, again accompanied by unspecific background. For both species, we report two-photon cross sections, *σ*_2*hv*_, since one photon is not sufficient over most of the studied range to remove the argon atom from ArFeH^+^ and ArFeD^+^, with a calculated binding energy of 3760 cm^−1^ on the CCSD/aug-cc-pVTZ level.^23^ One-photon cross sections are shown for comparison in the ESI,† Fig. S1.

**Table 1:** Experimental band positions and full-width at half maximum (FWHM) of Fe–H first overtone for ArFeH^+^, ArFeD^+^, Ar_2_FeH^+^, and Ar_2_FeD^+^, extracted from Fig. 1, along with theoretical comparison on the B3LYP-D3/aug-cc-pVTZ level (all in cm^−1^). For the harmonic value, the fundamental frequency was multiplied by 2 and an empirical scaling factor of 0.96 was applied. The anharmonic value is unscaled

	Experiment			Theory			
	Position	FWHM	D/H ratio	Harmonic	D/H ratio	Anharmonic	D/H ratio
ArFeH^+^	3636	55	0.720	3679	0.714	3725	0.720
ArFeD^+^	2618	62		2626		2682	
Ar_2_FeH^+^	3659	25	0.724	3659	0.714	3689	0.721
Ar_2_FeD^+^	2650	27		2611		2659	

The spectrum of Ar_2_FeH^+^, Fig. 1c, presents an intense band at 3659 ± 13 cm^−1^ together with weakly structured absorptions that cover almost the entire spectral range. Here, the Gaussian fits also yield very broad peaks that are most likely of electronic origin, summarized in Table S2 (ESI†). The IR spectrum of Ar_2_FeD^+^ in Fig. 1d exhibits an intense band at 2650 ± 14 cm^−1^, overlapping with five bands indexed in Tables S1 and S2 (ESI†). Interestingly, the second argon atom leads to a blue shift in the experiment. The effect is, however, not very pronounced, with a frequency change on the order of 1%.

To aid the interpretation of the experimental results and confirm the assignments of the Fe–H/D stretch overtones, we performed anharmonic vibrational frequency calculations at several levels of theory, see Table 1 and Tables S3–S10 (ESI†). In the following, B3LYP-D3/aug-cc-pVTZ values are reported. The assignment of the most intense band in Fig. 1a at 3636 cm^−1^ to the overtone of the Fe–H stretching mode in ArFeH^+^ is consistent with the calculated frequency of 3725 cm^−1^. The two weak resonances at 3690 cm^−1^ and 3755 cm^−1^ can be tentatively assigned to a combination band of the overtone of the Fe–H stretching mode with the Ar–Fe–H bending modes and the Fe–Ar stretching mode, respectively. Unfortunately, the anharmonic calculation with three vibrational quanta failed to provide physically reasonable values for these modes, thus we base this assignment on the addition of the overtone and fundamental frequencies of the respective modes. The feature at 2618 cm^−1^ in ArFeD^+^ present in Fig. 1b is again the first overtone of the Fe–D stretching mode, calculated at 2682 cm^−1^.

The experimental transition at 3659 cm^−1^ in Fig. 1c agrees well with the predicted anharmonic overtone at 3689 cm^−1^ of the Fe–H stretch in Ar_2_FeH^+^. The overtone band that corresponds to Ar_2_FeD^+^ in Fig. 1d is located at 2650 cm^−1^, which is in a good agreement with the calculated value of 2659 cm^−1^. A weak band at 3684 cm^−1^ for Ar_2_FeH^+^ hidden in the high frequency flank of the overtone is tentatively assigned to the combination band of the Fe–H overtone stretch with the Ar–Fe–Ar bending mode. A very weak potential combination band for Ar_2_FeD^+^ lies at 2777 cm^−1^, assignable to the Fe–D stretch overtone together with the Fe–Ar symmetric stretch. We tried to assign the equally weak band at 2543 cm^−1^ for Ar_2_FeD^+^, located 107 cm^−1^ below the Fe–D overtone, to a vibrational hot band, *i.e.* a (0,1) → (2,0) transition from excited Ar–Fe–Ar bending or stretching modes to the Fe–D stretch overtone, as indicated in Table S1 (ESI†). However, no convincing match was found.

For the linear structure of ArFeH^+^ and ArFeD^+^, the isotopic redshift of the overtone is 1018 cm^−1^, compared to 1009 cm^−1^ for Ar_2_FeH^+^ and Ar_2_FeD^+^, which corresponds to D/H wavenumber ratios of 0.720 and 0.724, respectively, consistent with the calculations, see Table 1. As noted above, the second argon atom induces an experimental blue shift of 23 cm^−1^ and 32 cm^−1^ for the Fe–H and Fe–D stretch overtone, respectively. While the calculations do not reproduce this blueshift, we note that the anharmonic calculations have severe difficulty handling the linear ArFeH^+^/ArFeD^+^ system. Since the shift is in the range of 1% of the vibrational frequencies, this rather seems to reflect the uncertainties of the calculations in these extremely anharmonic systems. However, the near-perfect agreement of the D/H wavenumber ratios, Table 1, underlines that the assignment of the peaks to the overtone of the Fe–H/D stretching mode is correct.

An interesting aspect is provided by the unusually large values for the FWHM of the overtone transitions in the linear ArFeH/D^+^ species. We previously rationalized the peak broadening of the Fe–H stretch fundamental in Ar_2_FeH^+^ by the shift of the Fe–H stretch as a function of the Ar–Fe–Ar angle.^23^ We repeated this analysis for the Ar–Fe–H bending mode, see ESI,† Fig. S6. The vibrational levels populated at 80 K extend to *v* = 3, and the Fe–H stretching mode shifts from 1916 cm^−1^ to ∼1900 cm^−1^. This means, the overtone covers a range of roughly 30 cm^−1^ due to the Ar–Fe–H bending mode. To account for rotational broadening, we performed a pGopher^55^ simulation of the rovibrational overtone spectrum of ArFeH^+^ at 80 K, Fig. S7a (ESI†), which we broadened with Gaussians of 30 cm^−1^, Fig. S7b (ESI†). The final broadened spectrum has a Gaussian line shape with FWHM of 32 cm^−1^, still somewhat narrower than the experimental spectrum. This means that our ions leave the ion source with a vibrational excitation closer to room temperature than to 80 K. We simulated radiative cooling of ArFeH^+^ thermalized at 300 K in a black-body radiation environment of 80 K, using our recently developed master equation modeling.^44,56,57^ Indeed, the ions need several seconds to lose a substantial part of their initial internal energy, see Fig. S8 (ESI†). We therefore attribute the large line width of the ArFeH^+^ overtone to the thermal energy of the ions, which lies between 80 K and room temperature.

**Scheme 1:**
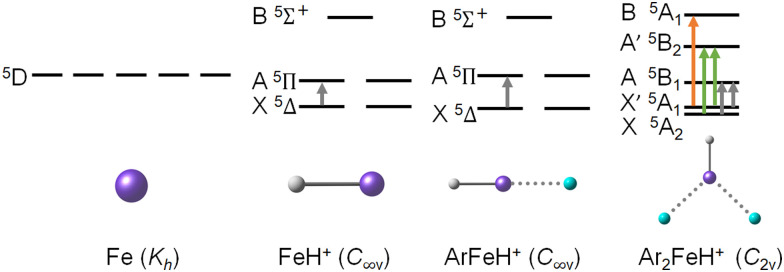
Splitting of the ^5^D term in iron upon addition of H^+^ and complexation with Ar. Symmetry allowed transitions from the lowest-lying states are shown with arrows. Spin–orbit coupling is not shown for clarity.

To understand the electronic contributions to the Ar_2_FeH^+^/Ar_2_FeD^+^ spectra, we analyze the symmetry breaking along the Fe–FeH^+^–ArFeH^+^–Ar_2_FeH^+^ series, Scheme 1, considering only the electronic states correlated with the ^5^D term in the Fe atom (the second lowest-lying term, ^5^F, lies at ∼7000 cm^−1^). When a proton is attached to the Fe ion, symmetry is reduced from *K*_h_ to *C*_∞v_, splitting the ^5^D term into X^5^Δ, A^5^Π and B^5^Σ^+^ molecular terms, with only one symmetry-allowed transition from the ground state (^5^Δ → ^5^Π). Upon addition of an argon atom, the system keeps its *C*_∞v_ symmetry, and only the relative energy of the terms changes somewhat. However, the second argon atom in Ar_2_FeH^+^ reduces symmetry further to *C*_2v_, producing X^5^A_2_, X′^5^A_1_, A^5^B_1_, A′^5^B_2_, and B^5^A_1_ molecular terms. Due to the low symmetry and the extensive mixing of X^5^A_2_ and X′^5^A_1_ terms, several allowed transitions arise. Note that spin–orbit effects are not shown for clarity, since their inclusion would overcrowd the scheme.

To analyze the origin of the broad bands in the overtone spectra, the spectral shape of electronic transitions to low-lying excited states of FeH^+^, ArFeH^+^ and Ar_2_FeH^+^ were modeled through reflection principle and MRCI(8,7)+SO/aug-cc-pVQZ calculations. To give an idea of the complexity of the spin–orbit states included in these calculations, we show potential curves along the Fe–H coordinate of the low-lying electronic states in Ar_2_FeH^+^ including spin–orbit coupling in Fig. S3 (ESI†).

**Fig. 2:**
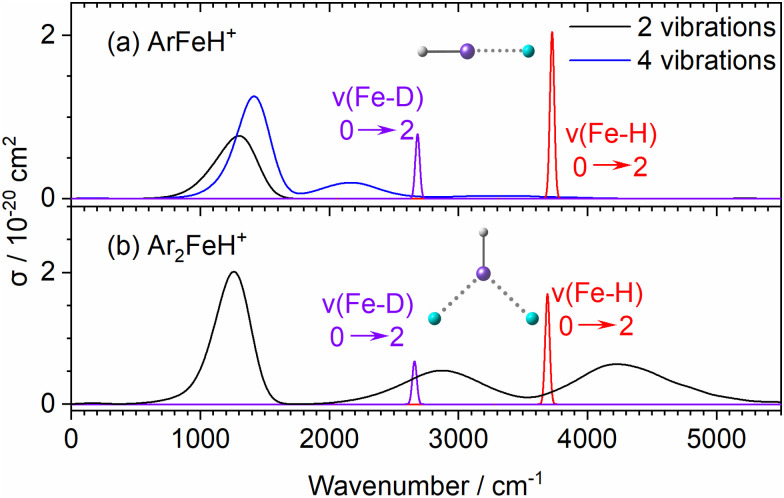
Theoretical absorption cross-section calculation for the electronic transition of (a) ArFeH^+^ and (b) Ar_2_FeH^+^ compared with the overtone of Fe–H/Fe–D vibrational stretching mode. For ArFeH^+^, structure sampling with only stretch vibrations (“2 vibrations”) and all frequencies (“4 vibrations”) were used, see text. Calculations were performed at the MRCI(8,7)+SO/aug-cc-pVQZ level of theory employing reflection principle modelling, with anharmonic overtone frequencies (shown in purple and red) at the B3LYP-D3/aug-cc-pVDZ level, broadened by Gaussians with the FWHM of 40 cm^−1^.

In the ArFeH^+^ complex, the electronic transition from X^5^Δ ground state to A^5^Π is predicted at around 1300 cm^−1^, with a calculated absorption cross section of ∼1 × 10^−20^ cm^2^, Fig. 2a. In the harmonic approximation sampling considering only two stretch vibrations (“2 vibrations”), this is the only peak of considerable intensity originating from electronic transitions below 5000 cm^−1^. When the linearity of the molecule is broken by including the bending modes in the modeling (“4 vibrations”), a second band appears at approximately 2000 cm^−1^ with cross section below 10^−21^ cm^2^. As the bending vibrations are strongly anharmonic, the actual spectrum is probably even broader.

In Ar_2_FeH^+^, Fig. 2b, the X^5^A_2_/X′^5^A_1_ → A^5^B_1_ band at 1300 cm^−1^ has virtually the same structure as in ArFeH^+^. Two additional bands appear in the experimentally studied range due to the lower symmetry. Their intensity is considerably higher compared to the second band in ArFeH^+^. The broad bands centered at 2635 cm^−1^ and 3088 cm^−1^ for Ar_2_FeH^+^ in Fig. 1c can be assigned to the X^5^A_2_/X′^5^A_1_ → A′^5^B_2_ transitions. The corresponding bands for Ar_2_FeD^+^ are observed at 2483 cm^−1^ and 3038 cm^−1^, respectively. However, no significant change in the structure of the electronic spectrum was observed for the deuterated species.

The cross sections for the electronic absorption in the simulated Ar_2_FeH^+^ and Ar_2_FeD^+^ spectra, Fig. 2b, are very similar to the values calculated for the Fe–H/D overtone transitions *v* = 0 → 2, consistent with the experimental spectra shown in Fig. 1c and d. In Fig. 2a, the weak broad bands emerging due to breaking of the linearity of ArFeH^+^/ArFeD^+^ may explain the unspecific background observed experimentally. Our results thus experimentally confirm the predictions of low-lying electronic states in FeH^+^ by Sodupe *et al.*, Langhoff *et al.* and Cheng and DeYonker.^20–22^

**Fig. 3:**
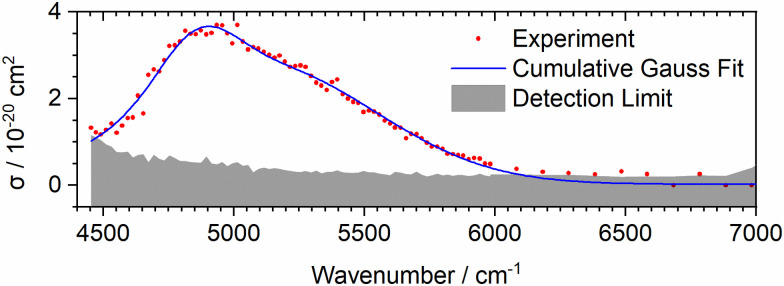
Experimental spectrum of the electronic transition B ← X′ of Ar_2_FeH^+^ in the 4450–7000 cm^−1^ range.

The additional band in the electronic spectrum of Ar_2_FeH^+^ predicted beyond 4000 cm^−1^, X′^5^A_1_ → B^5^A_1_, lies outside the range covered by Fig. 1. To validate the prediction, we performed further spectral measurements up to 14 000 cm^−1^, see Fig. 3 and Fig. S4 (ESI†). The predicted band is indeed there, with a cross section close to the value predicted in Fig. 2. While the main band is predicted by theory at 3500–5000 cm^−1^, we observe it shifted to the blue by about 1000 cm^−1^ (0.12 eV), within the expected accuracy of the electronically excited state calculations. With the reduced symmetry in Ar_2_FeH^+^, transitions to low-lying electronically excited states thus become spectroscopically accessible.

## Conclusion

In this study, we present a detailed theoretical and experimental investigation on the vibrational overtone transition of argon-tagged FeH^+^ and FeD^+^ and electronic transitions to low-lying excited states using IRMPD spectroscopy coupled with high-level quantum chemical calculations. We observed the overtone transition (*ν* = 0 → 2) of Fe–H stretching in Ar_2_FeH^+^, ArFeH^+^, and their deuterated isotopologues. According to the comparison between experiment and theory, the Fe–H fundamental stretching and its first overtone in bare FeH^+^ is expected in the 1790–1840 cm^−1^ and 3525–3619 cm^−1^ region, respectively.

Electronic transitions to the low-lying B^5^Σ^+^ state in FeH^+^ are symmetry forbidden. The second argon atom permanently reduces the *C*_∞v_ symmetry of FeH^+^ and ArFeH^+^ to *C*_2v_ in Ar_2_FeH^+^. This enhances the intensity of the transitions to low-lying excited states to about 10^−20^ cm^2^, making them fully accessible for IRMPD spectroscopy.

## Data availability

The data supporting this article have been included as part of the ESI.†

## Conflicts of interest

There are no conflicts of interest to declare.

## Supplementary Material

CP-026-D4CP03270E-s001
