# Supplementary material for: Symmetry reduction induced by argon tagging gives access to low-lying excited states of FeH+ in the overtone region of the Fe–H stretching mode
Source: Phys Chem Chem Phys. 2024 Oct 1;26(41):26363–9. doi: 10.1039/d4cp03270e (PMC11465007; doi:10.1039/d4cp03270e)
Supplement: CP-026-D4CP03270E-s001 [file CP-026-D4CP03270E-s001.pdf]

Supplementary Information (SI) for Physical Chemistry Chemical Physics.
This journal is © the Owner Societies 2024

Supporting Information

for

Symmetry reduction induced by argon tagging gives access to low-lying excited states of FeH^+ in the overtone region of the Fe-H stretching mode

Shan Jin,[a] *Marcos Juanes,*[a,b] *Christian van der Linde,*[a] *Milan Ončák,*[a]* *and Martin K. Beyer*[a]*

[a]Institut für Ionenphysik und Angewandte Physik, Universität Innsbruck, Technikerstraße 25, 6020 Innsbruck, Austria

[b]Departamento Química Física y Química Inorgánica, University of Valladolid, Paseo de Belén 7, 47011 Valladolid, Spain

Corresponding Authors

*E-mail: milan.oncak@uibk.ac.at; martin.beyer@uibk.ac.at

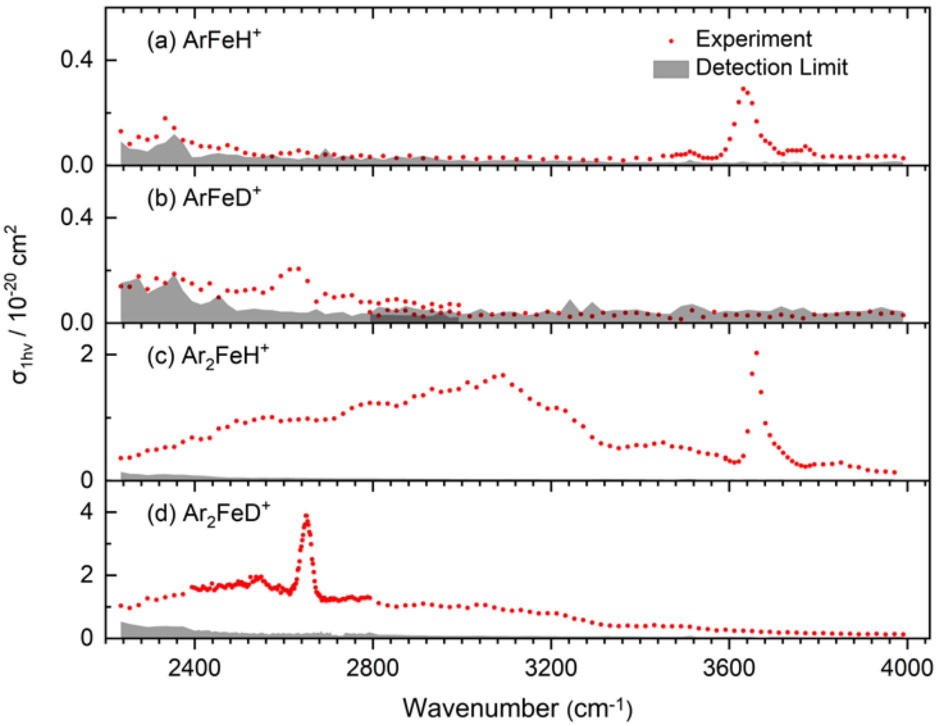

Figure S1. One-photon cross sections of experimental IRMPD spectra of a) $ArFeH^+$, b) $ArFeD^+$, c) Ar_2FeH^+, and d) Ar_2FeD^+, at T ≈ 80 K.

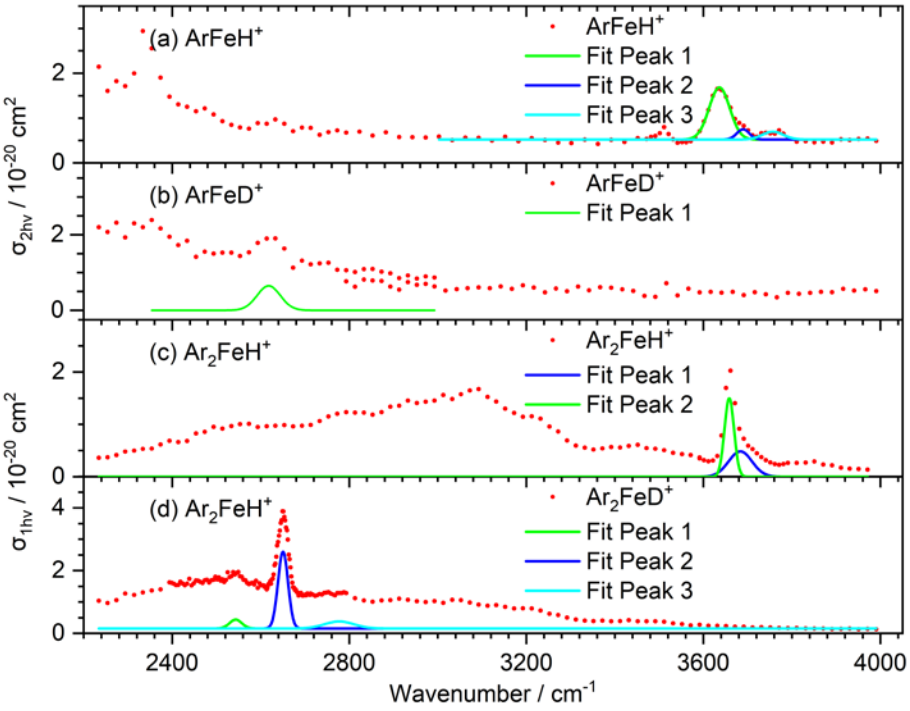

Figure S2. Potential vibrational contributions to the cumulative Gauss fit in Figure 1.

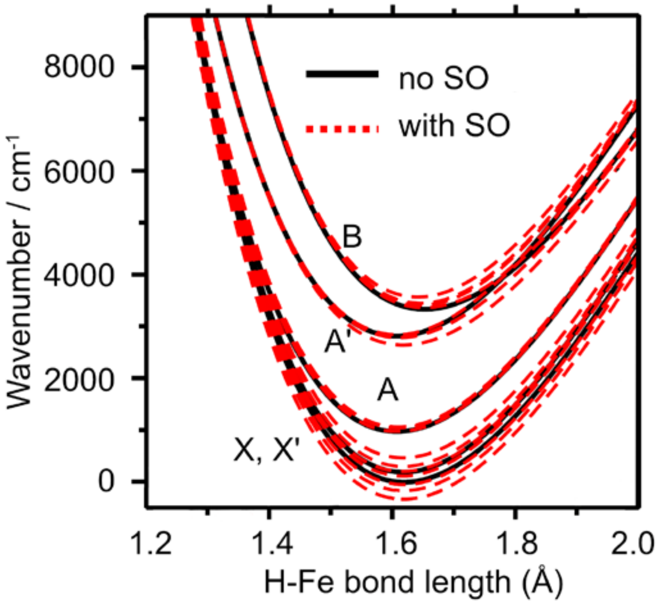

Figure S3. The simulated potential energy curves of Ar_2FeH^+ with, and without spin-orbital coupling performed at the MRCI(8,7)+SO/aug-cc-pVQZ(Fe,H), ECP10MWB(Ar) level of theory. Other molecular parameters are fixed to the equilibrium geometry as obtained at the B3LYP+D3/aug-cc-pVTZ level: Fe–Ar bond length 2.578 Å, Ar–Fe–Ar angle 94.0°.

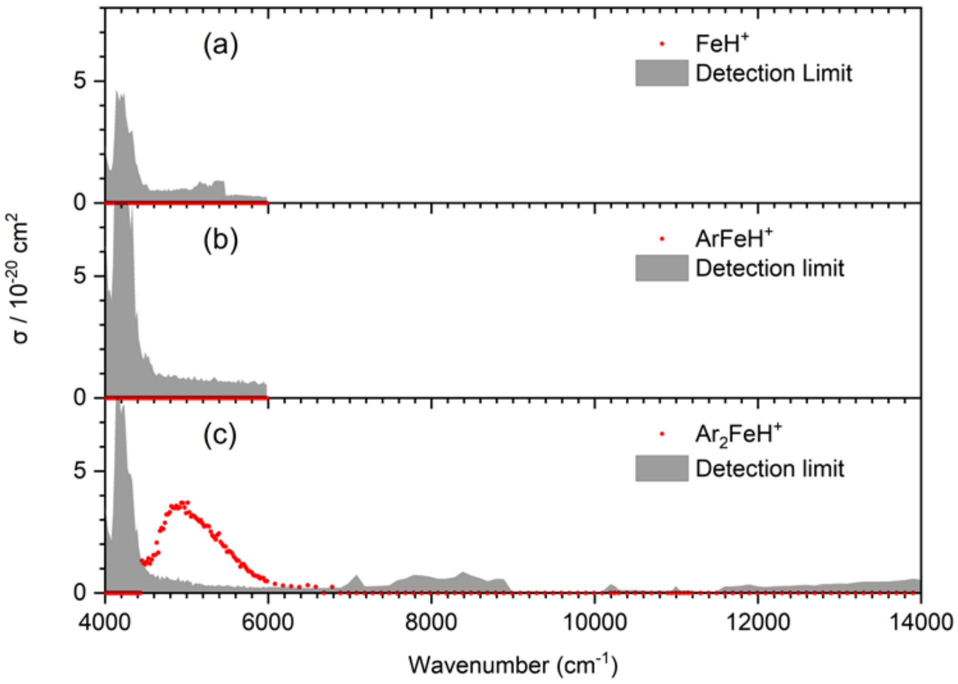

Figure S4. The infrared spectrum of FeH^+ and $ArFeH^+$ within the range of 4000 – 6000 cm^{-1}, and Ar_2FeH^+ within the range of 4000 - 14000 cm^{-1}. The scans for $ArFeH^+$ and FeH^+ were conducted in the region of 4000 – 6000 cm^{-1}, revealing no observable fragmentation. A prominent broad band for Ar_2FeH^+ is evident at approximately 5050 cm^{-1}, characterized by a FWHM of 890 cm^{-1}. It is noteworthy that the noise level within the 4000–4400 cm^{-1} range is affected by low laser energy.

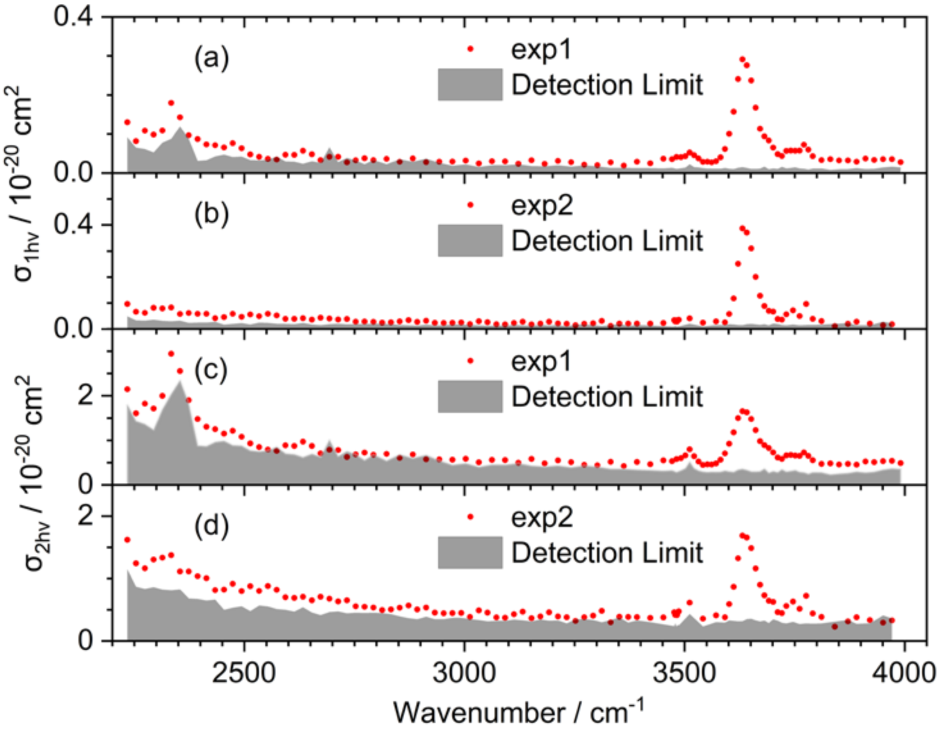

Figure S5. Additional experimental spectrum of $ArFeH^+$ (exp2) compared with the spectrum shown in Figure 1 (exp1). (a) and (b) cross sections assuming a one-photon process; (c) and (d) cross sections assuming a two-photon process.

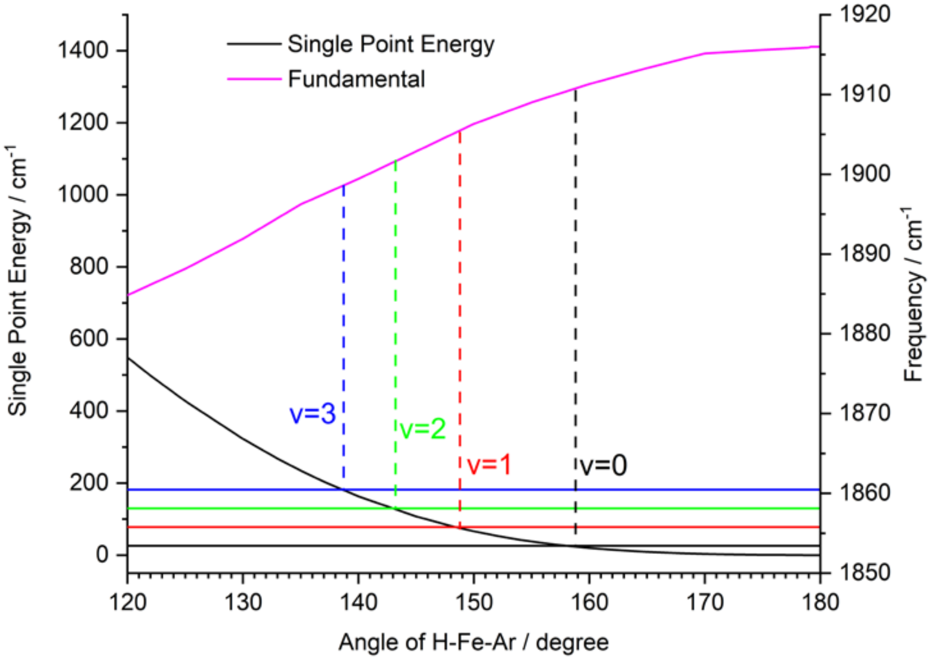

Figure S6. Single point energy for a relaxed scan of the H-Fe-Ar angle at the B3LYP-D3/aug-cc-pVTZ level and Fe–H stretch frequency calculated along the scan coordinate, with the option Freq=Projected in Gaussian. Classical minimum angles for thermally populated vibrational states at T = 80 K are labeled with dashed lines for better visualization. The harmonic frequency of the H-Fe-Ar bend of 52 cm^{-1}, as calculated at the same level of theory, was used for vibrational energy levels, which are marked by horizontal lines.

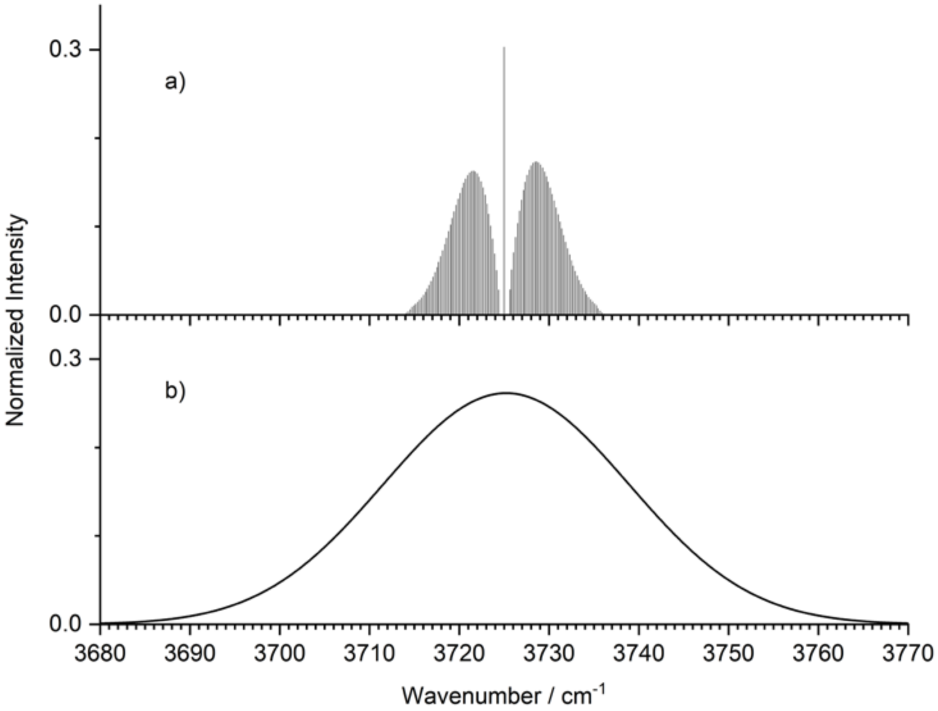

Figure S7. Ro-vibrational spectrum of $ArFeH^+$ simulated with pGopher at 80 K, assuming weak spin-orbital coupling. The molecular parameters were calculated at the B3LYP+D3/aug-cc-pVTZ level of theory within anharmonic vibrational analysis. a) No broadening; b) Gaussian broadening with FWHM of 30 cm^{-1}, resulting in a near-Gaussian peak with FWHM of 32 cm^{-1}.

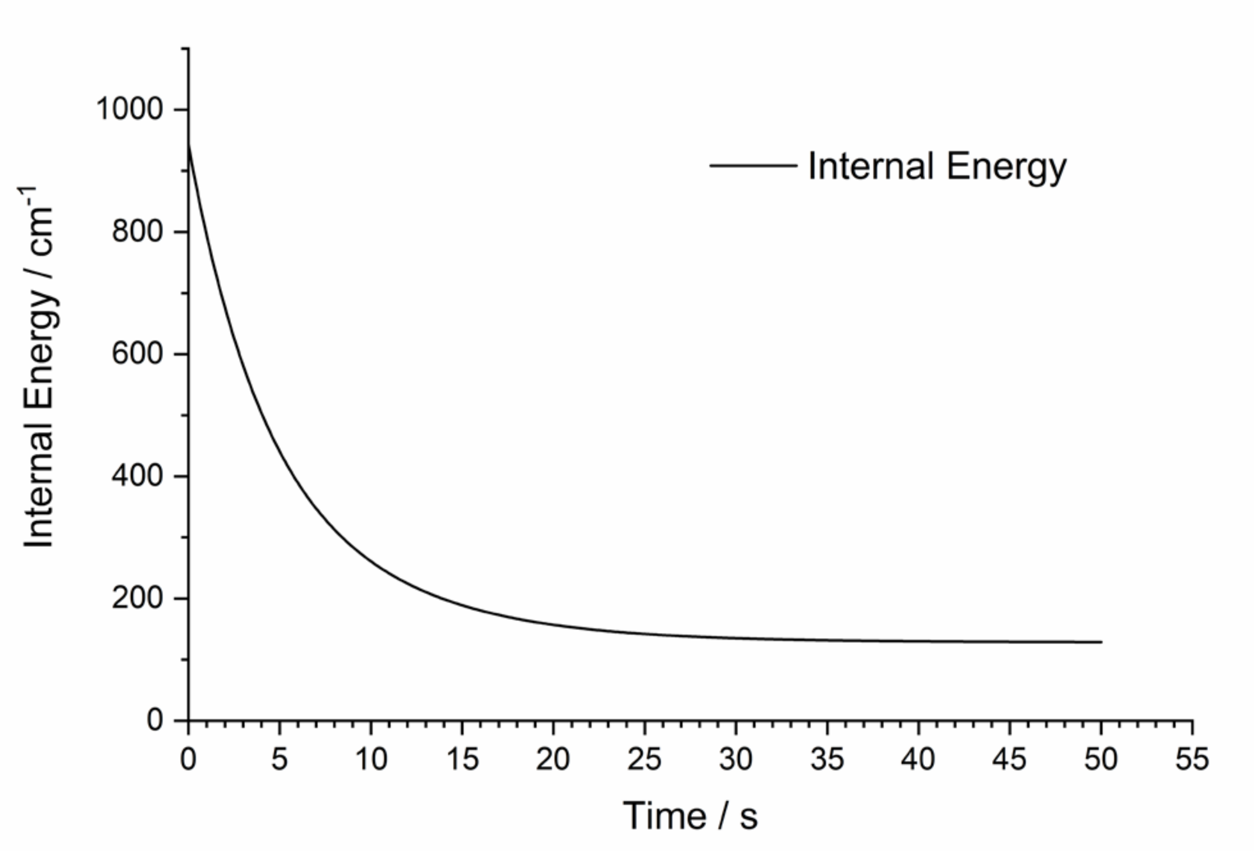

Figure S8. Radiative cooling curve of $ArFeH^+$ with initial temperature 300 K in a black-body environment at T = 80 K.

Table S1. Experimental band position *ν* and full-width at half maximum (FWHM) (in cm^{-1}) for $ArFeH^+$, $ArFeD^+$, Ar_2FeH^+, and Ar_2FeD^+ extracted from Figure 1. Calculated anharmonic vibrational frequencies (cm^{-1}) in the Fe–H overtone region are given for comparison, calculated using anharmonic frequency analysis at the B3LYP-D3/aug-cc-pVTZ level.

Ion	*ν*	FWHM	vibrational mode	symmetry	theory
**$ArFeH^+$**	3636	55	Fe-H stretch overtone	Σ^+	3725
	3690	33	Combination band of Fe-H stretch overtone and Ar-Fe-H bend	Π	*3776*[a]
	3757	55	Combination band of Fe-H stretch overtone and Fe-Ar stretch	Σ^+	*3900*[b]
**$ArFeD^+$**	2618	62	Fe-D stretch overtone	Σ^+	2682
**Ar_2FeH^+**	3659	25	Fe-H stretch overtone	A_1	3689
	3684	62	Combination band of Fe-H stretch overtone and Ar-Fe-Ar bend	A_1	3742[c]
**Ar_2FeD^+**	2543	33	Hot band of excited Ar-Fe-Ar bend to Fe-D stretch overtone	A_1	*2612*[b]
			Hot band of excited Fe-Ar antisymmetric stretch to Fe-D stretch overtone	B_2	*2513*[b]
			Hot band of excited Fe-Ar symmetric stretch to Fe-D stretch overtone	A_1	*2506*[b]
	2650	27	Fe-D stretch overtone	A_1	2659
	2777	77	Combination band of Fe-D stretch overtone and Fe-Ar symmetric stretch	A_1	*2810*[b]

[a] Fe–H overtone and harmonic fundamentals summed up as the anharmonic calculation provides unrealistic values for the Ar-Fe-H bend.

[b] Overtone and anharmonic fundamentals summed up / subtracted.

[c] Extracted from 3-quanta anharmonic calculation.

Table S2. Electronic transitions of Ar_2FeH^+ and Ar_2FeD^+ as extracted from Figure 1 and 3 (in cm^{-1}).

Ion	*ν*	FWHM
Ar_2FeH^+	2635	652
	3088	410
	3489	191
	3780	388
	4860	341
	5096	1051
Ar_2FeD^+	2483	497
	3038	455
	3504	238

Table S3. FeH^+ harmonic and anharmonic vibrational frequencies as calculated using various methods and the aug-cc-pVTZ basis set (in cm^{-1}). The frequencies are unscaled.

Mode	B3LYP	B3LYP-D3	CAM-B3LYP	*ω*B97XD	MP2	CCSD
Harmonic Fundamental	1879	1855	1928	1924	1952	1871
Anharmonic Fundamental	1847	1793	1899	1896	1935	
Anharmonic Overtone	3662	3525	3768	3763	3852	

Table S4. FeD^+ harmonic and anharmonic vibrational frequencies as calculated using various methods and the aug-cc-pVTZ basis set (in cm^{-1}). The frequencies are unscaled.

Mode	B3LYP	B3LYP-D3	CAM-B3LYP	*ω*B97XD	MP2	CCSD
Harmonic Fundamental	1341	1324	1376	1373	1393	1335
Anharmonic Fundamental	1325	1293	1361	1358	1384	
Anharmonic Overtone	2633	2554	2707	2702	2760	

Table S5. $ArFeH^+$ harmonic vibrational frequencies[a] and anharmonic vibrational frequencies[b] as calculated using various methods and the aug-cc-pVTZ basis set (in cm^{-1}). The frequencies are unscaled. For CAM-B3LYP and ωB97XD functionals, unphysical results were obtained in the anharmonic calculation, which are not shown.

symmetry	mode	B3LYP	B3LYP-D3	CAM-B3LYP	ωB97XD	MP2	CCSD
Π	H-Fe-Ar bend[a]	56	52	65	39	147	133
Σ^+	Fe-Ar stretch[a]	172	174	185	176	187	181
Σ^+	Fe-H stretch[a]	1918	1916	1959	1945	1930	1894
	Fe-H stretch[b]	1888	1881[c]	–	–	1891	–
Σ^+	Fe-H overtone[b]	3744	3725	–	–	3741	–

[c] Fixed symmetry causes deviation from Table 2 in Jin et al., *J. Phys. Chem. Lett.* **13**, 5867–5872 (2022). DOI: 10.1021/acs.jpclett.2c01511.

Table S6. $ArFeD^+$ harmonic vibrational frequencies[a] and anharmonic vibrational frequencies[b] as calculated using various methods and the aug-cc-pVTZ basis set (in cm^{-1}). The frequencies are unscaled.

symmetry	mode	B3LYP	B3LYP-D3	CAM-B3LYP	ωB97XD	MP2	CCSD
Π	D-Fe-Ar bend[a]	41	37	47	27	107	94
Σ^+	Fe-Ar stretch[a]	171	173	184	175	186	181
Σ^+	Fe-D stretch[a]	1369	1367	1398	1388	1377	1353
	Fe-D stretch[b]	1373	1350	1384	1429	1358	
Σ^+	Fe-D overtone[b]	2730	2682	2753	2851	2696	

Table S7. Ar_2FeH^+ harmonic vibrational frequencies as calculated using various methods and the aug-cc-pVTZ basis set. The frequencies are unscaled.[a]

symmetry	mode	B3LYP	B3LYP-D3	CAM-B3LYP	ωB97XD	MP2
A_1	Fe-Ar bend	43	46	45	47	54
B_2	Fe-Ar asymmetric stretch	148	151	160	146	147
A_1	Fe-Ar symmetric stretch	152	157	165	151	166
B_1	Fe-H bend out of the plane	207	206	214	207	209
B_2	Fe-H bend in the plane	237	234	245	236	211
A_1	Fe-H stretch	1906	1906	1947	1931	1924

[a] All values reproduced from Table S3 in Jin et al., *J. Phys. Chem. Lett.* **13**, 5867–5872 (2022). DOI: 10.1021/acs.jpclett.2c01511.

Table S8. Ar_2FeH^+ anharmonic vibrational frequencies as calculated using various methods and the aug-cc-pVTZ basis set. The frequencies are unscaled.

symmetry	mode	B3LYP	B3LYP-D3	CAM-B3LYP	ωB97XD	MP2
A_1	Fe-Ar bend	43	50	56	42	29
B_2	Fe-Ar asymmetry stretch	139	144	158	212	198
A_1	Fe-Ar symmetry stretch	147	153	163	206	151
B_1	Fe-H bend out of the plane	202	208	275	284	229
B_2	Fe-H bend in the plane	231	235	298	245	253
A_1	Fe-H stretch	1868 [a]	1864 [a]	1918 [a]	1988 [a]	1882 [a]
A_1	Fe-H overtone	3703	3689	3806	4008	3721

[a] Fixed symmetry causes deviations from Table S4 in Jin et al., *J. Phys. Chem. Lett.* **13**, 5867–5872 (2022). DOI: 10.1021/acs.jpclett.2c01511.

Table S9. Ar_2FeD^+ harmonic vibrational frequencies (in cm^{-1}) as calculated using various methods and the aug-cc-pVTZ basis set. The frequencies are unscaled.

symmetry	mode	B3LYP	B3LYP-D3	CAM-B3LYP	ωB97XD	MP2
A_1	Fe-Ar bend	43	46	45	47	54
B_2	Fe-Ar asymmetry stretch	148	151	160	146	139
A_1	Fe-Ar symmetry stretch	152	157	165	150	165
B_1	Fe-D bend out of the plane	152	151	157	151	153
B_2	Fe-D bend in the plane	169	168	175	169	160
A_1	Fe-D stretch	1360	1360	1390	1377	1373

Table S10. Ar_2FeD^+ anharmonic vibrational frequencies (in cm^{-1}) as calculated using various methods and the aug-cc-pVTZ basis set. The frequencies are unscaled.

symmetry	mode	B3LYP	B3LYP-D3	CAM-B3LYP	ωB97XD	MP2
A_1	Fe-Ar bend	43	46	53	41	36
B_2	Fe-Ar asymmetry stretch	141	145	157	219	158
A_1	Fe-Ar symmetry stretch	146	152	162	207	154
B_1	Fe-D bend out of the plane	141	152	195	189	179
B_2	Fe-D bend in the plane	159	169	212	165	257
A_1	Fe-D stretch	1341	1339	1376	1408	1353
A_1	Fe-D overtone	2665	2659	2736	2832	2684

Experimental spectral data

Table S11. $ArFeH^+$ 1&2-photon process cross-section data

Wavenumber	ArFeH+	Detection limit	ArFeH+	Detection limit
cm^{-1}	1-photon cross-section		2-photon cross-section	
2234.087	1.30E-21	9.15E-22	2.15E-20	1.79E-20
2254.049	8.18E-22	6.49E-22	1.61E-20	1.43E-20
2274.010	1.08E-21	6.14E-22	1.82E-20	1.36E-20
2293.972	9.81E-22	5.28E-22	1.71E-20	1.24E-20
2313.934	1.09E-21	7.80E-22	2.00E-20	1.69E-20
2333.895	1.79E-21	8.78E-22	2.94E-20	2.04E-20
2353.857	1.43E-21	1.20E-21	2.55E-20	2.34E-20
2373.818	9.65E-22	8.76E-22	1.90E-20	1.81E-20
2393.780	8.71E-22	3.15E-22	1.48E-20	8.82E-21
2413.742	7.31E-22	3.30E-22	1.31E-20	8.67E-21
2433.703	7.12E-22	4.25E-22	1.25E-20	9.59E-21
2453.665	6.54E-22	4.73E-22	1.15E-20	9.83E-21
2473.626	7.65E-22	4.12E-22	1.21E-20	8.88E-21
2493.588	6.32E-22	4.21E-22	1.08E-20	8.78E-21
2513.550	4.74E-22	3.32E-22	9.32E-21	7.72E-21
2533.511	4.13E-22	3.25E-22	8.48E-21	7.51E-21
2553.473	3.62E-22	3.42E-22	7.93E-21	7.69E-21
2573.434	3.45E-22	4.24E-22	7.66E-21	8.52E-21
2593.396	4.66E-22	2.97E-22	8.88E-21	7.01E-21
2613.358	4.63E-22	2.88E-22	8.73E-21	6.84E-21
2633.319	5.69E-22	2.36E-22	9.70E-21	6.17E-21
2653.281	4.79E-22	3.02E-22	8.73E-21	6.91E-21
2673.242	3.29E-22	2.59E-22	7.07E-21	6.30E-21
2693.204	4.14E-22	6.63E-22	7.90E-21	1.01E-20
2713.166	4.16E-22	2.80E-22	7.81E-21	6.38E-21
2733.127	2.74E-22	3.89E-22	6.27E-21	7.55E-21
2753.089	3.25E-22	3.23E-22	6.85E-21	6.78E-21
2773.050	3.77E-22	2.23E-22	7.23E-21	5.55E-21
2793.012	3.28E-22	3.32E-22	6.74E-21	6.74E-21
2822.954	3.60E-22	2.24E-22	6.95E-21	5.42E-21
2852.897	2.89E-22	3.55E-22	6.08E-21	6.79E-21
2882.839	3.74E-22	2.88E-22	6.81E-21	5.95E-21
2912.782	2.75E-22	3.71E-22	5.75E-21	6.69E-21
2942.724	2.87E-22	2.59E-22	5.71E-21	5.45E-21
2972.666	2.92E-22	1.87E-22	5.63E-21	4.48E-21

Wavenumber	ArFeH+	Detection limit	ArFeH+	Detection limit
cm^{-1}	1-photon cross-section		2-photon cross-section	
3002.609	3.17E-22	2.18E-22	5.86E-21	4.82E-21
3032.551	2.44E-22	1.53E-22	5.10E-21	4.02E-21
3062.494	2.99E-22	2.00E-22	5.63E-21	4.56E-21
3092.436	2.96E-22	1.99E-22	5.58E-21	4.53E-21
3122.378	2.37E-22	2.21E-22	4.97E-21	4.83E-21
3152.321	3.25E-22	1.78E-22	5.83E-21	4.29E-21
3182.263	2.33E-22	1.69E-22	4.82E-21	4.08E-21
3212.206	3.10E-22	1.99E-22	5.50E-21	4.34E-21
3242.148	2.75E-22	1.62E-22	5.13E-21	3.90E-21
3272.090	2.10E-22	1.96E-22	4.52E-21	4.34E-21
3302.033	2.00E-22	1.70E-22	4.42E-21	4.06E-21
3331.975	2.73E-22	1.28E-22	5.18E-21	3.55E-21
3361.918	1.87E-22	1.32E-22	4.22E-21	3.55E-21
3391.860	2.87E-22	1.25E-22	5.17E-21	3.37E-21
3421.802	2.46E-22	1.27E-22	4.65E-21	3.28E-21
3451.745	3.67E-22	1.19E-22	5.51E-21	3.11E-21
3471.706	3.53E-22	1.33E-22	5.32E-21	3.25E-21
3481.687	4.12E-22	1.04E-22	5.78E-21	2.85E-21
3491.668	4.06E-22	1.21E-22	6.00E-21	3.24E-21
3501.649	4.31E-22	1.38E-22	6.60E-21	3.68E-21
3511.630	5.24E-22	2.24E-22	7.99E-21	5.16E-21
3521.610	4.55E-22	1.52E-22	6.42E-21	3.68E-21
3531.591	3.73E-22	1.41E-22	5.25E-21	3.19E-21
3541.572	2.83E-22	1.24E-22	4.46E-21	2.93E-21
3551.553	2.87E-22	1.12E-22	4.60E-21	2.83E-21
3561.534	2.72E-22	1.15E-22	4.57E-21	2.93E-21
3571.514	3.00E-22	1.09E-22	4.84E-21	2.88E-21
3581.495	4.01E-22	1.10E-22	5.65E-21	2.92E-21
3591.476	6.12E-22	1.29E-22	7.08E-21	3.18E-21
3601.457	1.00E-21	1.11E-22	9.23E-21	2.98E-21
3611.438	1.57E-21	1.04E-22	1.18E-20	2.89E-21
3621.418	2.41E-21	1.33E-22	1.50E-20	3.36E-21
3631.399	2.91E-21	1.50E-22	1.65E-20	3.55E-21
3641.380	2.76E-21	1.28E-22	1.63E-20	3.32E-21
3651.361	2.37E-21	1.07E-22	1.48E-20	2.97E-21
3661.342	1.67E-21	1.08E-22	1.23E-20	2.97E-21
3671.322	1.13E-21	1.15E-22	9.82E-21	3.02E-21
3681.303	9.39E-22	1.61E-22	8.92E-21	3.59E-21

Wavenumber	ArFeH+	Detection limit	ArFeH+	Detection limit
cm^{-1}	1-photon cross-section		2-photon cross-section	
3691.284	8.33E-22	9.56E-23	8.28E-21	2.70E-21
3701.265	6.48E-22	1.19E-22	7.27E-21	3.04E-21
3711.246	4.84E-22	1.05E-22	6.15E-21	2.79E-21
3721.226	4.35E-22	1.57E-22	5.83E-21	3.46E-21
3731.207	5.65E-22	1.17E-22	6.61E-21	2.95E-21
3741.188	5.74E-22	1.29E-22	6.72E-21	3.10E-21
3751.169	5.67E-22	1.47E-22	6.56E-21	3.26E-21
3761.150	5.71E-22	1.17E-22	6.50E-21	2.88E-21
3771.130	7.24E-22	1.11E-22	7.30E-21	2.76E-21
3781.111	5.98E-22	8.99E-23	6.49E-21	2.44E-21
3791.092	4.32E-22	1.14E-22	5.53E-21	2.79E-21
3811.054	3.27E-22	1.17E-22	4.72E-21	2.79E-21
3831.015	3.52E-22	8.17E-23	4.83E-21	2.30E-21
3850.977	3.19E-22	9.70E-23	4.60E-21	2.49E-21
3870.938	3.21E-22	1.03E-22	4.72E-21	2.63E-21
3890.900	2.90E-22	1.25E-22	4.56E-21	2.95E-21
3910.862	3.68E-22	1.04E-22	5.27E-21	2.73E-21
3930.823	3.35E-22	1.11E-22	5.11E-21	2.91E-21
3950.785	3.52E-22	1.42E-22	5.33E-21	3.36E-21
3970.746	3.52E-22	1.64E-22	5.40E-21	3.66E-21
3990.708	2.77E-22	1.43E-22	4.91E-21	3.51E-21

Table S12. $ArFeD^+$ 1&2-photon process cross-section data

Wavenumber	ArFeD+	Detection limit	ArFeD+	Detection limit
cm^{-1}	1-photon cross-section		2-photon cross-section	
2234.087	1.40E-21	1.54E-21	2.20E-20	2.31E-20
2254.049	1.38E-21	1.60E-21	2.07E-20	2.24E-20
2274.010	1.77E-21	1.72E-21	2.32E-20	2.29E-20
2293.972	1.28E-21	1.13E-21	1.93E-20	1.80E-20
2313.934	1.70E-21	1.32E-21	2.30E-20	2.02E-20
2333.895	1.51E-21	1.48E-21	2.20E-20	2.17E-20
2353.857	1.87E-21	1.88E-21	2.39E-20	2.39E-20
2373.818	1.65E-21	1.23E-21	2.16E-20	1.86E-20
2393.780	1.50E-21	8.36E-22	1.94E-20	1.45E-20
2413.742	1.27E-21	7.13E-22	1.73E-20	1.28E-20
2433.703	1.50E-21	8.10E-22	1.84E-20	1.34E-20
2453.665	9.81E-22	1.07E-21	1.41E-20	1.49E-20
2473.626	1.23E-21	7.05E-22	1.55E-20	1.17E-20
2493.588	1.19E-21	4.72E-22	1.50E-20	9.28E-21
2513.550	1.24E-21	5.05E-22	1.52E-20	9.58E-21
2533.511	1.29E-21	5.49E-22	1.53E-20	9.83E-21
2553.473	1.15E-21	5.07E-22	1.43E-20	9.34E-21
2573.434	1.32E-21	4.96E-22	1.53E-20	9.23E-21
2593.396	1.79E-21	4.34E-22	1.79E-20	8.59E-21
2613.358	2.04E-21	4.40E-22	1.90E-20	8.63E-21
2633.319	2.06E-21	3.96E-22	1.90E-20	8.09E-21
2653.281	1.60E-21	3.98E-22	1.63E-20	7.99E-21
2673.242	8.12E-22	5.37E-22	1.13E-20	9.11E-21
2693.204	1.10E-21	3.41E-22	1.31E-20	7.18E-21
2713.166	9.76E-22	3.92E-22	1.22E-20	7.64E-21
2733.127	1.02E-21	4.18E-22	1.24E-20	7.84E-21
2753.089	1.06E-21	2.67E-22	1.26E-20	6.22E-21
2773.050	8.01E-22	3.73E-22	1.07E-20	7.27E-21
2793.012	7.95E-22	3.72E-22	1.06E-20	7.17E-21
2812.974	7.45E-22	2.98E-22	1.01E-20	6.32E-21
2832.935	8.73E-22	3.15E-22	1.09E-20	6.44E-21
2852.897	9.04E-22	2.98E-22	1.10E-20	6.19E-21
2872.858	8.14E-22	2.92E-22	1.02E-20	6.05E-21
2892.820	7.60E-22	3.03E-22	9.79E-21	6.10E-21
2912.782	5.98E-22	2.66E-22	8.49E-21	5.61E-21
2932.743	7.17E-22	2.69E-22	9.22E-21	5.53E-21
2952.705	6.21E-22	4.10E-22	8.39E-21	6.80E-21

Wavenumber	ArFeD+	Detection limit	ArFeD+	Detection limit
cm^{-1}	1-photon cross-section		2-photon cross-section	
2972.666	7.18E-22	2.09E-22	8.94E-21	4.74E-21
2992.628	6.80E-22	2.36E-22	8.64E-21	5.01E-21
2793.012	4.17E-22	3.83E-22	7.64E-21	7.32E-21
2812.974	2.88E-22	6.20E-22	6.22E-21	9.20E-21
2832.935	4.91E-22	5.68E-22	8.13E-21	8.74E-21
2852.897	4.73E-22	4.67E-22	7.88E-21	7.82E-21
2872.858	4.73E-22	6.57E-22	7.76E-21	9.20E-21
2892.820	3.17E-22	5.60E-22	6.25E-21	8.35E-21
2912.782	2.50E-22	5.07E-22	5.46E-21	7.84E-21
2932.743	4.70E-22	4.99E-22	7.42E-21	7.64E-21
2952.705	3.98E-22	3.97E-22	6.74E-21	6.69E-21
2972.666	4.43E-22	4.86E-22	6.97E-21	7.32E-21
2992.628	3.66E-22	3.36E-22	6.31E-21	6.01E-21
3017.580	2.58E-22	3.79E-22	5.21E-21	6.39E-21
3042.532	2.97E-22	5.47E-22	5.57E-21	7.66E-21
3067.484	3.26E-22	3.20E-22	5.85E-21	5.80E-21
3092.436	3.47E-22	4.26E-22	6.04E-21	6.71E-21
3117.388	3.25E-22	4.37E-22	5.83E-21	6.80E-21
3142.340	3.74E-22	2.90E-22	6.30E-21	5.52E-21
3167.292	3.17E-22	3.66E-22	5.66E-21	6.08E-21
3192.244	4.31E-22	4.95E-22	6.59E-21	7.09E-21
3217.196	3.79E-22	3.41E-22	6.01E-21	5.70E-21
3242.148	2.39E-22	9.11E-22	4.75E-21	9.46E-21
3267.100	3.42E-22	4.02E-22	5.75E-21	6.24E-21
3292.052	2.58E-22	8.06E-22	5.06E-21	9.07E-21
3317.004	3.94E-22	4.36E-22	6.25E-21	6.61E-21
3341.956	2.89E-22	4.97E-22	5.29E-21	7.00E-21
3366.908	3.88E-22	4.90E-22	6.11E-21	6.90E-21
3391.860	3.82E-22	5.26E-22	5.99E-21	7.05E-21
3416.812	3.48E-22	4.29E-22	5.55E-21	6.20E-21
3441.764	3.15E-22	3.57E-22	5.11E-21	5.45E-21
3466.716	1.85E-22	3.52E-22	3.84E-21	5.35E-21
3491.668	1.48E-22	6.44E-22	3.57E-21	7.58E-21
3516.620	4.80E-22	7.26E-22	7.14E-21	8.88E-21
3541.572	2.25E-22	5.74E-22	3.93E-21	6.38E-21
3566.524	4.22E-22	3.75E-22	5.72E-21	5.38E-21
3591.476	2.97E-22	4.70E-22	4.76E-21	6.06E-21

Wavenumber	ArFeD+	Detection limit	ArFeD+	Detection limit
cm^{-1}	1-photon cross-section		2-photon cross-section	
3616.428	3.10E-22	4.40E-22	4.89E-21	5.84E-21
3641.380	2.84E-22	5.08E-22	4.67E-21	6.32E-21
3666.332	3.11E-22	4.66E-22	4.89E-21	6.04E-21
3691.284	2.22E-22	4.59E-22	4.04E-21	5.85E-21
3716.236	3.17E-22	6.38E-22	4.79E-21	6.89E-21
3741.188	2.57E-22	4.80E-22	4.23E-21	5.86E-21
3766.140	1.79E-22	5.90E-22	3.43E-21	6.38E-21
3791.092	3.28E-22	3.59E-22	4.62E-21	4.86E-21
3816.044	3.05E-22	3.77E-22	4.37E-21	4.87E-21
3840.996	3.34E-22	3.98E-22	4.61E-21	5.04E-21
3865.948	3.67E-22	4.66E-22	4.87E-21	5.51E-21
3890.900	3.35E-22	4.49E-22	4.79E-21	5.56E-21
3915.852	4.38E-22	4.88E-22	5.67E-21	6.00E-21
3940.804	3.52E-22	6.08E-22	5.21E-21	6.92E-21
3965.756	3.84E-22	4.92E-22	5.57E-21	6.33E-21
3990.708	3.00E-22	4.48E-22	5.07E-21	6.25E-21

Table S13. Ar_2FeH^+ 1-photon process cross-section data

Wavenumber	Ar2FeH+	Detection limit
cm^{-1}	1-photon cross-section	
2234.087	3.59E-21	1.42E-21
2254.049	3.68E-21	1.14E-21
2274.010	4.12E-21	1.09E-21
2293.972	4.79E-21	9.17E-22
2313.934	4.95E-21	1.05E-21
2333.895	5.29E-21	1.08E-21
2353.857	5.37E-21	1.00E-21
2373.818	6.16E-21	9.93E-22
2393.780	6.85E-21	8.94E-22
2413.742	6.60E-21	8.45E-22
2433.703	6.81E-21	7.45E-22
2453.665	8.26E-21	6.41E-22
2473.626	8.56E-21	5.57E-22
2493.588	9.50E-21	5.21E-22
2513.550	9.18E-21	5.38E-22
2533.511	9.67E-21	4.70E-22
2553.473	1.00E-20	5.27E-22
2573.434	1.01E-20	4.77E-22
2593.396	9.46E-21	4.96E-22
2613.358	9.63E-21	5.07E-22
2633.319	9.72E-21	4.57E-22
2653.281	9.84E-21	4.56E-22
2673.242	9.60E-21	4.44E-22
2693.204	9.71E-21	4.38E-22
2713.166	9.86E-21	3.96E-22
2733.127	1.06E-20	3.92E-22
2753.089	1.15E-20	4.10E-22
2773.050	1.20E-20	3.85E-22
2793.012	1.23E-20	3.82E-22
2812.974	1.22E-20	3.72E-22
2832.935	1.22E-20	3.53E-22
2852.897	1.19E-20	3.67E-22
2872.858	1.23E-20	3.51E-22
2892.820	1.34E-20	3.30E-22
2912.782	1.36E-20	3.38E-22
2932.743	1.45E-20	3.01E-22
2952.705	1.41E-20	3.00E-22

Wavenumber	Ar2FeH+	Detection limit
cm^{-1}	1-photon cross-section	
2972.666	1.43E-20	2.98E-22
2992.628	1.45E-20	2.82E-22
3012.590	1.56E-20	2.75E-22
3032.551	1.48E-20	2.63E-22
3052.513	1.58E-20	2.58E-22
3072.474	1.65E-20	2.55E-22
3092.436	1.67E-20	2.87E-22
3112.398	1.52E-20	2.76E-22
3132.359	1.44E-20	2.65E-22
3152.321	1.30E-20	2.65E-22
3172.282	1.22E-20	2.41E-22
3192.244	1.14E-20	2.40E-22
3212.206	1.15E-20	2.28E-22
3232.167	1.11E-20	2.31E-22
3252.129	9.56E-21	2.34E-22
3272.090	8.62E-21	2.21E-22
3292.052	6.91E-21	2.56E-22
3312.014	5.93E-21	2.71E-22
3331.975	5.54E-21	2.67E-22
3351.937	5.18E-21	2.64E-22
3371.898	5.40E-21	2.46E-22
3391.860	5.66E-21	2.31E-22
3411.822	5.57E-21	2.39E-22
3431.783	5.89E-21	2.01E-22
3451.745	6.08E-21	1.91E-22
3471.706	5.45E-21	1.86E-22
3491.668	5.34E-21	2.22E-22
3511.630	5.07E-21	2.96E-22
3531.591	4.53E-21	1.79E-22
3551.553	4.30E-21	1.74E-22
3571.514	4.10E-21	1.75E-22
3591.476	3.48E-21	2.10E-22
3591.476	3.67E-21	1.88E-22
3601.457	3.14E-21	2.14E-22
3611.438	2.92E-21	1.94E-22
3621.418	3.08E-21	1.90E-22
3631.399	3.93E-21	2.07E-22
3641.380	7.85E-21	2.14E-22

Wavenumber	Ar2FeH+	Detection limit
cm^{-1}	1-photon cross-section	
3651.361	1.70E-20	2.06E-22
3661.342	2.03E-20	2.12E-22
3671.322	1.41E-20	1.84E-22
3681.303	9.13E-21	2.02E-22
3691.284	7.24E-21	2.13E-22
3701.265	5.90E-21	2.05E-22
3711.246	5.27E-21	1.96E-22
3721.226	4.45E-21	1.92E-22
3731.207	3.72E-21	1.84E-22
3741.188	3.13E-21	1.91E-22
3751.169	2.70E-21	1.81E-22
3761.150	2.40E-21	1.99E-22
3771.130	2.26E-21	2.13E-22
3781.111	2.42E-21	2.09E-22
3791.092	2.61E-21	2.08E-22
3811.054	2.62E-21	1.98E-22
3831.015	2.68E-21	2.03E-22
3850.977	2.88E-21	2.07E-22
3870.938	2.31E-21	2.05E-22
3890.900	2.15E-21	2.21E-22
3910.862	1.70E-21	2.58E-22
3930.823	1.51E-21	2.62E-22
3950.785	1.48E-21	2.38E-22
3970.746	1.35E-21	2.60E-22

Table S14. Ar_2FeD^+ 1-photon process cross-section data

Wavenumber	Ar2FeD+	Detection limit
cm^{-1}	1-photon cross-section	
2234.087	1.04E-20	5.31E-21
2254.049	9.63E-21	4.61E-21
2274.010	1.05E-20	4.10E-21
2293.972	1.27E-20	3.64E-21
2313.934	1.22E-20	3.87E-21
2333.895	1.30E-20	3.97E-21
2353.857	1.37E-20	3.83E-21
2373.818	1.40E-20	3.81E-21
2393.780	1.63E-20	2.78E-21
2398.770	1.61E-20	2.54E-21
2403.761	1.58E-20	2.50E-21
2408.751	1.54E-20	2.74E-21
2413.742	1.54E-20	2.42E-21
2418.732	1.64E-20	2.53E-21
2423.722	1.59E-20	2.54E-21
2428.713	1.53E-20	2.66E-21
2433.703	1.58E-20	2.55E-21
2438.694	1.74E-20	1.98E-21
2443.684	1.53E-20	2.26E-21
2448.674	1.59E-20	2.24E-21
2453.665	1.69E-20	2.01E-21
2458.655	1.68E-20	1.97E-21
2463.646	1.64E-20	2.07E-21
2468.636	1.61E-20	2.09E-21
2473.626	1.69E-20	1.96E-21
2478.617	1.71E-20	1.78E-21
2483.607	1.62E-20	1.94E-21
2488.598	1.69E-20	1.88E-21
2493.588	1.71E-20	2.04E-21
2497.580	1.80E-20	1.79E-21
2501.573	1.72E-20	1.65E-21
2505.565	1.78E-20	2.00E-21
2509.557	1.68E-20	1.71E-21
2513.550	1.72E-20	1.73E-21
2517.542	1.63E-20	1.72E-21
2521.534	1.70E-20	1.61E-21
2525.526	1.94E-20	1.57E-21

Wavenumber	Ar2FeD+	Detection limit
cm^{-1}	1-photon cross-section	
2529.519	1.79E-20	1.61E-21
2533.511	1.82E-20	1.81E-21
2537.503	1.95E-20	1.49E-21
2541.496	1.88E-20	1.52E-21
2545.488	1.92E-20	1.67E-21
2549.480	1.96E-20	1.88E-21
2553.473	1.82E-20	1.79E-21
2557.465	1.80E-20	1.43E-21
2561.457	1.71E-20	1.57E-21
2565.450	1.69E-20	1.29E-21
2569.442	1.61E-20	1.35E-21
2573.434	1.57E-20	1.70E-21
2577.427	1.59E-20	1.67E-21
2581.419	1.61E-20	1.46E-21
2585.411	1.56E-20	1.65E-21
2589.404	1.68E-20	1.67E-21
2593.396	1.45E-20	1.47E-21
2597.388	1.60E-20	1.71E-21
2601.381	1.53E-20	1.71E-21
2605.373	1.53E-20	1.41E-21
2607.369	1.52E-20	1.61E-21
2609.365	1.47E-20	1.65E-21
2611.361	1.46E-20	1.60E-21
2613.358	1.40E-20	1.74E-21
2615.354	1.45E-20	1.55E-21
2617.350	1.57E-20	1.54E-21
2619.346	1.56E-20	1.48E-21
2621.342	1.66E-20	1.40E-21
2623.338	1.58E-20	1.34E-21
2625.334	1.71E-20	1.63E-21
2627.331	1.74E-20	1.62E-21
2629.327	1.87E-20	1.64E-21
2631.323	2.17E-20	1.73E-21
2633.319	2.22E-20	1.90E-21
2635.315	2.44E-20	1.49E-21
2637.311	2.80E-20	1.55E-21
2639.308	2.91E-20	1.35E-21
2641.304	3.11E-20	1.54E-21

Wavenumber	Ar2FeD+	Detection limit
cm^{-1}	1-photon cross-section	
2643.300	3.28E-20	1.49E-21
2645.296	3.52E-20	1.60E-21
2647.292	3.60E-20	1.68E-21
2649.288	3.89E-20	1.59E-21
2651.285	3.88E-20	1.95E-21
2653.281	3.71E-20	1.77E-21
2655.277	3.60E-20	1.50E-21
2657.273	3.37E-20	1.75E-21
2659.269	3.28E-20	1.61E-21
2661.265	2.98E-20	1.51E-21
2663.262	2.51E-20	1.62E-21
2665.258	2.37E-20	1.83E-21
2667.254	2.10E-20	2.10E-21
2669.250	1.81E-20	1.66E-21
2671.246	1.61E-20	1.79E-21
2673.242	1.46E-20	1.67E-21
2675.238	1.45E-20	1.87E-21
2677.235	1.39E-20	1.98E-21
2679.231	1.39E-20	1.63E-21
2681.227	1.32E-20	1.84E-21
2683.223	1.30E-20	2.45E-21
2685.219	1.20E-20	1.96E-21
2687.215	1.21E-20	1.61E-21
2689.212	1.27E-20	1.56E-21
2691.208	1.29E-20	1.55E-21
2693.204	1.23E-20	1.48E-21
2695.200	1.20E-20	1.83E-21
2697.196	1.21E-20	1.95E-21
2699.192	1.23E-20	1.73E-21
2701.189	1.21E-20	1.65E-21
2703.185	1.22E-20	1.65E-21
2705.181	1.27E-20	2.18E-21
2707.177	1.27E-20	1.04E-21
2709.173	1.25E-20	1.08E-21
2711.169	1.22E-20	1.04E-21
2713.166	1.25E-20	1.12E-21
2713.166	1.27E-20	1.24E-21
2718.156	1.23E-20	1.12E-21

Wavenumber	Ar2FeD+	Detection limit
cm^{-1}	1-photon cross-section	
2723.146	1.19E-20	8.44E-22
2728.137	1.22E-20	9.25E-22
2733.127	1.25E-20	9.74E-22
2738.118	1.23E-20	1.82E-21
2743.108	1.29E-20	1.71E-21
2748.098	1.30E-20	1.52E-21
2753.089	1.32E-20	1.50E-21
2758.079	1.27E-20	1.71E-21
2763.070	1.24E-20	1.41E-21
2768.060	1.21E-20	1.58E-21
2773.050	1.28E-20	1.59E-21
2778.041	1.28E-20	1.51E-21
2783.031	1.28E-20	1.43E-21
2788.022	1.31E-20	1.83E-21
2793.012	1.28E-20	1.91E-21
2812.974	1.12E-20	1.19E-21
2832.935	1.04E-20	1.22E-21
2852.897	1.00E-20	1.17E-21
2872.858	1.05E-20	1.06E-21
2892.820	1.06E-20	9.71E-22
2912.782	1.10E-20	1.02E-21
2932.743	1.07E-20	8.50E-22
2952.705	1.02E-20	8.44E-22
2972.666	9.94E-21	7.95E-22
2992.628	1.03E-20	8.09E-22
3012.590	9.70E-21	7.99E-22
3032.551	1.07E-20	8.92E-22
3052.513	1.05E-20	8.52E-22
3072.474	9.81E-21	7.77E-22
3092.436	9.14E-21	8.17E-22
3112.398	8.80E-21	6.90E-22
3132.359	8.97E-21	7.68E-22
3152.321	8.44E-21	8.27E-22
3172.282	7.90E-21	7.46E-22
3192.244	7.96E-21	7.33E-22
3212.206	7.94E-21	7.33E-22
3232.167	7.24E-21	6.46E-22
3252.129	6.27E-21	6.75E-22

Wavenumber	Ar2FeD+	Detection limit
cm^{-1}	1-photon cross-section	
3272.090	5.86E-21	7.32E-22
3292.052	5.03E-21	6.51E-22
3312.014	4.41E-21	7.01E-22
3331.975	4.05E-21	6.88E-22
3351.937	3.89E-21	6.62E-22
3371.898	4.05E-21	7.05E-22
3391.860	3.88E-21	6.37E-22
3411.822	3.91E-21	5.61E-22
3431.783	4.21E-21	5.36E-22
3451.745	3.88E-21	5.61E-22
3471.706	3.86E-21	5.35E-22
3491.668	3.85E-21	5.46E-22
3511.630	3.71E-21	8.36E-22
3531.591	3.20E-21	4.81E-22
3551.553	2.93E-21	4.78E-22
3571.514	2.55E-21	5.12E-22
3591.476	2.74E-21	4.91E-22
3611.438	2.44E-21	5.07E-22
3631.399	2.45E-21	4.52E-22
3651.361	2.35E-21	4.80E-22
3671.322	2.06E-21	2.70E-22
3691.284	2.08E-21	3.87E-22
3711.246	1.82E-21	4.37E-22
3731.207	1.92E-21	3.87E-22
3751.169	1.82E-21	4.08E-22
3771.130	1.67E-21	3.52E-22
3791.092	1.60E-21	3.52E-22
3811.054	1.66E-21	3.84E-22
3831.015	1.55E-21	3.53E-22
3850.977	1.49E-21	3.96E-22
3870.938	1.46E-21	3.92E-22
3890.900	1.32E-21	3.91E-22
3910.862	1.39E-21	4.04E-22
3930.823	1.47E-21	4.75E-22
3950.785	1.25E-21	4.94E-22
3970.746	1.37E-21	5.36E-22
3990.708	1.27E-21	5.71E-22

Table S15. Ar_2FeH^+ 1-photon cross-section in 4450-14000 cm^{-1} range.

Wavenumber	Ar2FeH+	Detection limit
cm^{-1}	1-photon cross-section	
4453.803	1.33E-20	1.16E-20
4471.636	1.22E-20	1.11E-20
4491.110	1.17E-20	1.04E-20
4511.807	1.28E-20	9.42E-21
4532.095	1.42E-20	8.93E-21
4551.709	1.21E-20	7.64E-21
4572.626	1.37E-20	7.62E-21
4593.863	1.55E-20	7.72E-21
4613.953	1.56E-20	6.41E-21
4633.177	2.06E-20	7.01E-21
4653.643	1.66E-20	7.14E-21
4673.478	2.55E-20	5.34E-21
4693.721	2.67E-20	7.04E-21
4714.179	2.62E-20	6.11E-21
4733.950	2.88E-20	5.73E-21
4753.871	3.22E-20	6.93E-21
4774.145	3.23E-20	6.26E-21
4794.372	3.31E-20	5.61E-21
4814.738	3.56E-20	5.56E-21
4834.330	3.49E-20	5.35E-21
4854.562	3.48E-20	5.26E-21
4874.546	3.57E-20	5.21E-21
4895.134	3.48E-20	6.66E-21
4914.957	3.52E-20	5.03E-21
4935.267	3.70E-20	4.82E-21
4955.576	3.69E-20	5.37E-21
4975.421	3.51E-20	4.42E-21
4994.486	3.28E-20	5.09E-21
5014.572	3.70E-20	5.33E-21
5034.631	3.31E-20	4.49E-21
5054.896	3.13E-20	4.68E-21
5074.419	3.19E-20	2.99E-21
5094.913	3.15E-20	3.78E-21
5115.584	3.07E-20	4.07E-21
5135.956	3.01E-20	3.74E-21
5155.931	2.94E-20	3.97E-21
5175.587	2.99E-20	3.76E-21

Wavenumber	Ar2FeH+	Detection limit
cm^{-1}	1-photon cross-section	
5195.847	2.85E-20	3.50E-21
5215.371	2.73E-20	3.31E-21
5235.238	2.75E-20	3.14E-21
5255.827	2.76E-20	3.04E-21
5275.241	2.73E-20	3.29E-21
5295.685	2.52E-20	2.96E-21
5315.906	2.37E-20	3.38E-21
5335.798	2.30E-20	2.78E-21
5356.060	2.20E-20	3.06E-21
5376.162	2.38E-20	3.11E-21
5396.386	2.44E-20	3.44E-21
5416.954	2.10E-20	2.98E-21
5437.439	2.00E-20	2.89E-21
5457.628	1.92E-20	2.87E-21
5477.213	1.90E-20	2.92E-21
5497.064	1.69E-20	3.31E-21
5517.798	1.72E-20	2.98E-21
5536.817	1.70E-20	2.97E-21
5557.374	1.63E-20	2.68E-21
5577.942	1.49E-20	2.52E-21
5598.599	1.42E-20	2.32E-21
5617.293	1.33E-20	3.11E-21
5638.625	1.33E-20	2.94E-21
5659.495	1.08E-20	2.59E-21
5678.917	1.19E-20	2.53E-21
5699.919	1.18E-20	3.13E-21
5719.250	1.08E-20	2.46E-21
5739.359	9.82E-21	2.05E-21
5758.335	8.90E-21	2.29E-21
5779.118	8.85E-21	2.67E-21
5799.536	8.34E-21	2.30E-21
5818.392	7.25E-21	2.47E-21
5838.898	7.16E-21	2.18E-21
5859.353	6.91E-21	2.39E-21
5878.439	6.80E-21	2.34E-21
5899.148	5.95E-21	2.13E-21
5920.635	6.25E-21	2.29E-21
5942.265	6.13E-21	2.67E-21

Wavenumber	Ar2FeH+	Detection limit
cm^{-1}	1-photon cross-section	
5962.002	4.95E-21	2.27E-21
5981.147	4.89E-21	2.06E-21
5983.577	4.88E-21	2.50E-21
6082.396	3.78E-21	2.54E-21
6182.988	3.08E-21	2.38E-21
6282.165	2.78E-21	2.39E-21
6383.279	2.46E-21	2.20E-21
6484.348	3.17E-21	1.94E-21
6582.602	2.52E-21	2.03E-21
6684.117	0	2.03E-21
6784.030	2.53E-21	2.29E-21
6883.629	0	2.16E-21
6982.153	0	4.01E-21
7082.484	0	7.59E-21
7181.220	0	2.78E-21
7282.595	0	2.83E-21
7381.376	0	2.98E-21
7481.575	0	3.19E-21
7580.995	0	5.27E-21
7681.972	0	6.00E-21
7781.653	0	7.39E-21
7881.071	0	7.22E-21
7980.840	0	6.66E-21
8080.808	0	5.88E-21
8181.867	0	5.93E-21
8282.856	0	6.53E-21
8384.261	0	8.87E-21
8483.441	0	7.21E-21
8584.929	0	6.24E-21
8685.879	0	4.91E-21
8785.501	0	5.80E-21
8886.061	0	5.79E-21
8986.833	0	7.45E-22
9087.712	0	7.02E-22
9188.052	0	8.59E-22
9289.484	0	7.16E-22
9390.165	0	7.17E-22
9491.538	0	6.38E-22

Wavenumber	Ar2FeH+	Detection limit
cm^{-1}	1-photon cross-section	
9591.379	0	6.23E-22
9692.703	0	6.07E-22
9792.689	0	5.57E-22
9892.107	0	6.78E-22
9992.156	0	6.01E-22
10092.530	0	5.28E-22
10194.290	0	3.71E-21
10243.180	0	2.96E-21
10296.060	0	1.29E-21
10395.320	0	1.20E-21
10495.590	0	1.21E-21
10595.500	0	1.03E-21
10694.550	0	8.48E-22
10794.940	0	1.05E-21
10894.560	0	8.72E-22
10943.400	0	9.27E-22
10994.880	0	2.95E-21
11044.220	0	1.05E-21
11095.360	0	9.64E-22
11145.020	0	1.16E-21
11195.960	0	7.31E-22
11297.140	0	7.68E-22
11395.690	0	7.18E-22
11494.900	0	6.41E-22
11593.890	0	2.79E-21
11693.240	0	2.74E-21
11794.480	0	3.07E-21
11895.230	0	3.59E-21
11993.920	0	2.80E-21
12093.290	0	2.56E-21
12193.750	0	2.79E-21
12294.810	0	2.92E-21
12395.380	0	2.97E-21
12494.960	0	3.22E-21
12596.250	0	3.35E-21
12695.780	0	3.12E-21
12796.710	0	3.73E-21
12895.370	0	3.75E-21

Wavenumber	Ar2FeH+	Detection limit
cm^{-1}	1-photon cross-section	
12995.370	0	3.97E-21
13094.820	0	3.81E-21
13195.360	0	4.34E-21
13295.610	0	4.73E-21
13396.740	0	4.65E-21
13497.880	0	4.76E-21
13599.060	0	4.75E-21
13700.860	0	5.05E-21
13801.970	0	5.34E-21
13903.100	0	5.90E-21
14006.070	0	5.49E-21
